# Novel Insights into The Roles of N^6^-methyladenosine (m^6^A) Modification and Autophagy in Human Diseases

**DOI:** 10.7150/ijbs.75466

**Published:** 2023-01-01

**Authors:** Jiaxin Liang, Jingwen Sun, Wei Zhang, Xiwen Wang, Ying Xu, Yuan Peng, Ling Zhang, Wenqian Xiong, Yi Liu, Hengwei Liu

**Affiliations:** 1 Department of Obstetrics and Gynecology, Union Hospital, Tongji Medical College, Huazhong University of Science and Technology, Wuhan 430022, China.; 2 Department of Obstetrics and Gynecology, Zhongnan Hospital of Wuhan University, Wuhan University, Wuhan 430071, China.

**Keywords:** RNA modification, N^6^-methyladenosine (m^6^A), Autophagy, Biomarkers, Therapeutic targets

## Abstract

Autophagy is an evolutionarily conserved cellular degradation and recycling process. It is important for maintaining vital cellular function and metabolism. Abnormal autophagy activity can cause the development of various diseases. N^6^-methyladenosine (m^6^A) methylation is the most prevalent and abundant internal modification in eukaryotes, affecting almost all aspects of RNA metabolism. The process of m^6^A modification is dynamic and adjustable. Its regulation depends on the regulation of m^6^A methyltransferases, m^6^A demethylases, and m^6^A binding proteins. m^6^A methylation and autophagy are two crucial and independent cellular events. Recent studies have shown that m^6^A modification mediates the transcriptional and post-transcriptional regulation of autophagy-related genes, affecting autophagy regulatory networks in multiple diseases. However, the regulatory effects of m^6^A regulators on autophagy in human diseases are not adequately acknowledged. In the present review, we summarized the latest knowledge of m^6^A modification in autophagy and elucidated the molecular regulatory mechanisms underlying m^6^A modification in autophagy regulatory networks. Moreover, we discuss the potentiality of m^6^A regulators serving as promising predictive biomarkers for human disease diagnosis and targets for therapy. This review will increase our understanding of the relationship between m^6^A methylation and autophagy, and provide novel insights to specifically target m^6^A modification in autophagy-associated therapeutic strategies.

## Introduction

The diverse coding, structural, and biological activities of RNA are based on several alterations 1. N^6^-methyladenosine (m^6^A) RNA methylation is the most prevalent and well-conserved post-transcriptional RNA modification in eukaryotes, which affects almost every aspect of RNA metabolisms, such as stability 2, 3, splicing 4, localization 5, translation efficiency 6, and RNA-protein interaction 7. m^6^A modification plays a crucial role in many physiological and pathological processes, such as ultraviolet (UV)-induced DNA damage repair 8, circadian rhythm control 9, stem cell development 10, tumor progression 11, and drug resistance. Autophagy is a self-phagocytosis and degradation process, which maintains the steady state of the cell by decomposing non-essential cell components. The abnormal activation of autophagy is closely associated with diverse pathologies, including cancer 12-15 and numerous benign diseases 16-19. Owing to the lack of present knowledge on the role of m^6^A RNA methylation in autophagy in human diseases, we aimed to summarize the effect of m^6^A methylation on autophagy regulatory elements in human diseases in this review and elucidated the underlying mechanisms of m^6^A-mediated autophagy modulating, which can be used as a potential novel technique in autophagy-associated diagnostic and therapeutic strategies.

### 1.1 m^6^A writers, erasers, and readers

**Figure 1:**
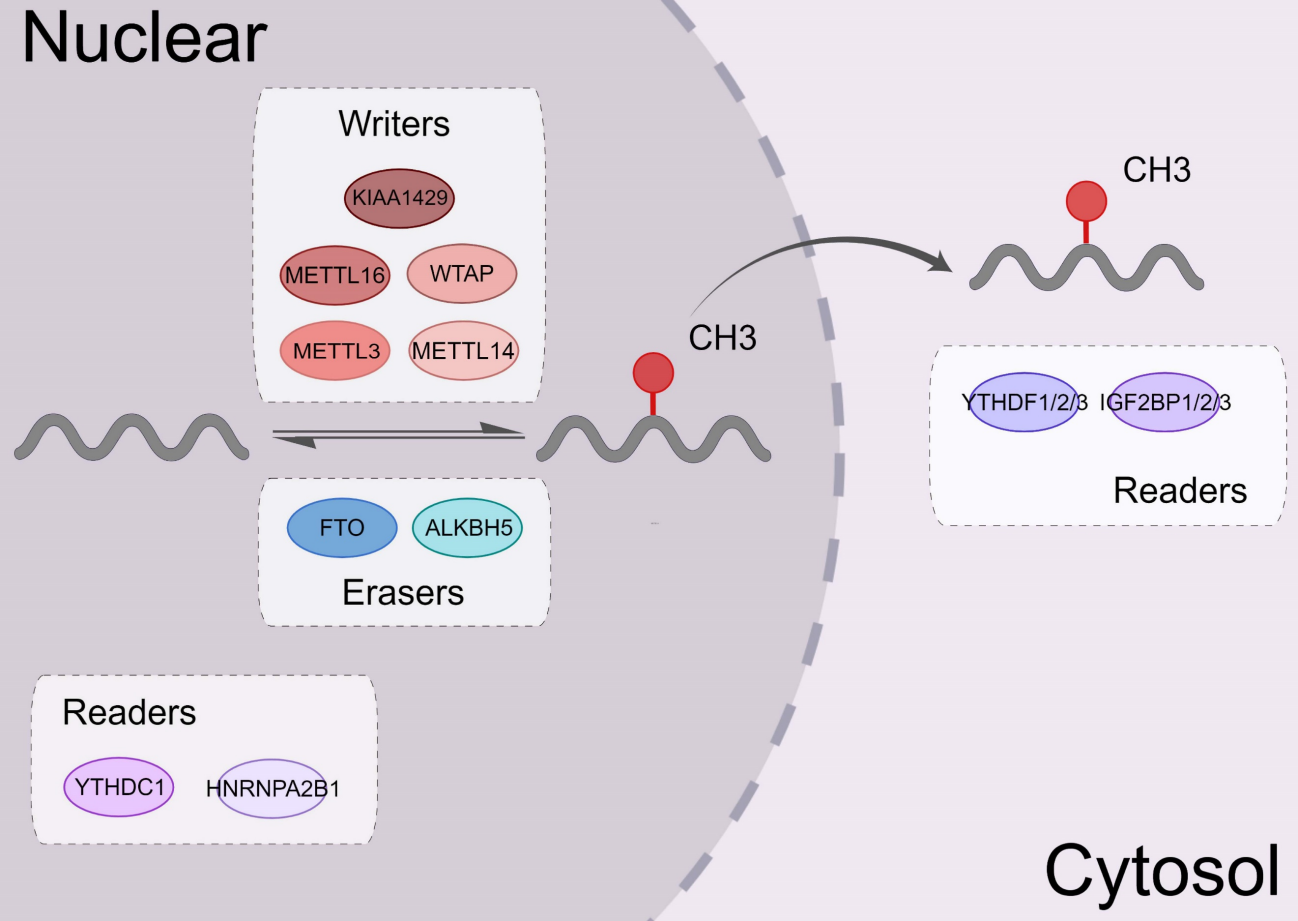
** The biological functions of m^6^A regulators.** m^6^A modifying enzymes include writers, erasers and readers. Writers (METTL3, METTL14, WTAP, KIAA1429, METTL16, etc.) can add a methyl group to different types of RNA. Erasers (ALKBH5 and FTO) can eliminate this modification. Readers (the YTH family, IGF2BP1, and hnRNPA2B1) could identify the m^6^A modification sites and regulate the downstream functional activities.

m^6^A modification is a dynamic and reversible process. Three complexes, namely m^6^A methyltransferases, m^6^A demethylases, and m^6^A binding proteins, participate in this dynamic regulation event 20 (Figure 1).

m^6^A methyltransferases, also known as "writers," catalyze the formation of N^6^-methyladenosine (m^6^A) by inserting a methyl substituent on the N atom of adenosine at position 6^21^. They include proteins, such as methyltransferase-like 3/14 (METTL3/14) 22, 23, Wilms tumor 1-associated protein (WTAP) 24, and KIAA1429 25. METTL14 binds with METTL3 to form a heterodimeric methyltransferase complex, that is extremely conserved in mammals 26. Among them, METTL3 is the catalytically active subunit and METTL14 mainly acts on substrate recognition with its degenerated catalytic center 23. WTAP is an important regulatory subunit of m^6^A methyltransferase complex. It can remarkably alter the level of m^6^A modification 27.

**Figure 2:**
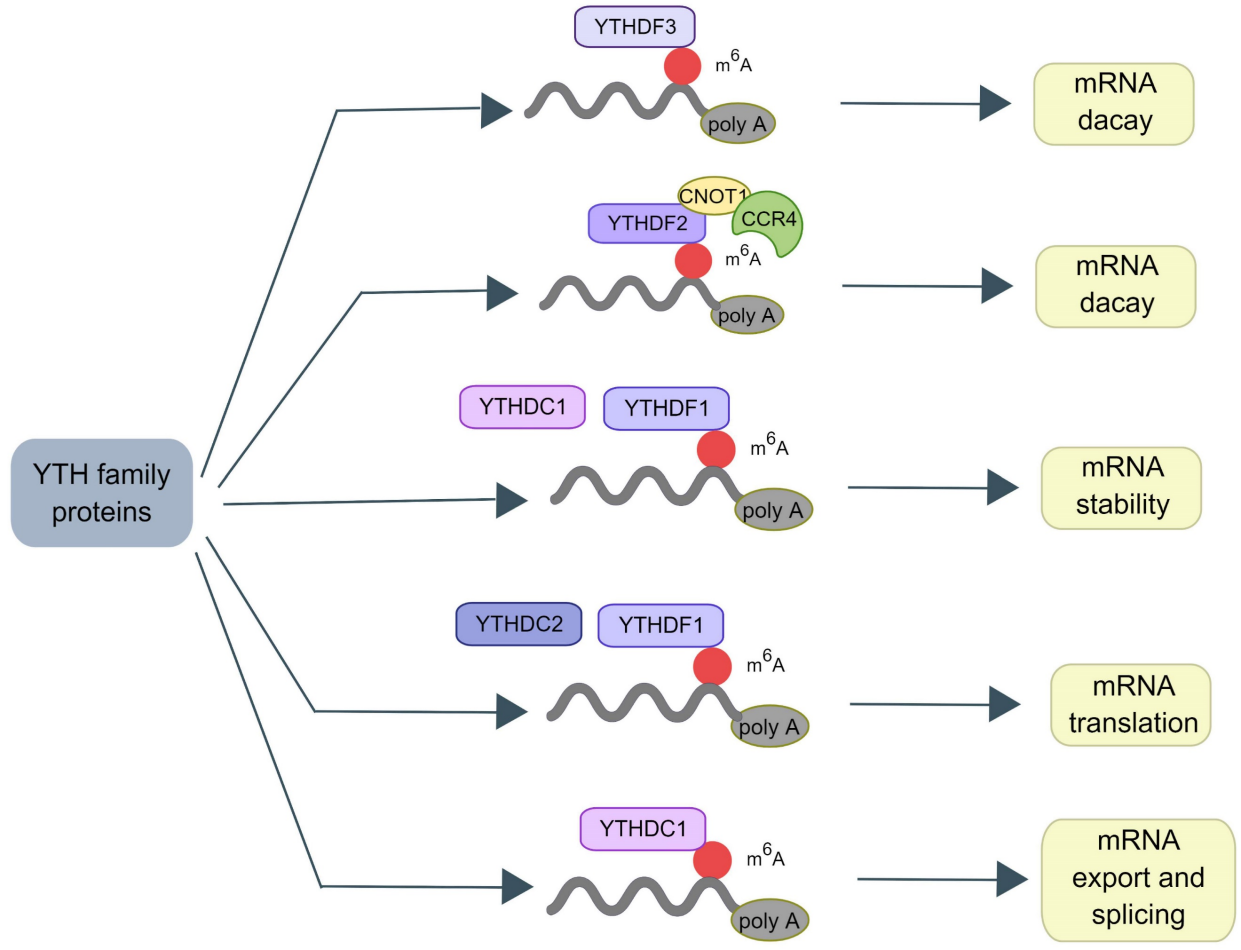
** The mechanism of the YTH family proteins.** In proteins containing the YTH domain, YTHDF2 promotes the decay of mRNA by recruiting the CCR4-NOT complex. YTHDF3 also helps mRNA decay. YTHDC2 and YTHDF1 can regulate the translation of mRNA. YTHDC1 can selectively splice pre-mRNA into mature transcripts and mediate the nuclear output of mature mRNA. Meanwhile, YTHDC1 can improve the stability of mRNA as well as YTHDF1.

Demethylases such as fat mass and obesity-associated protein (FTO) 28 and human AlkB Homolog H5 (ALKBH5) 29, served as “erasers” to selectively remove the m^6^A mark via several complex mechanisms, thereby affecting certain biological processes. The amount of m^6^A in the RNA life cycle was found to be steady in a previous study 30, and demethylases only operate in specific situations. FTO, as the first m^6^A demethylase discovered, is mainly enriched in nuclear speckles but not enriched in paraspeckles 31. The subcellular localization of FTO in cells determines its accessibility to different RNA substrates 32. The overexpression or knockdown of FTO decreased or increased the level of m^6^A modifications, respectively. ALKBH5 is one of the members of the AlkB family. ALKBH5 is dysregulated in most tissues and plays a vital role in various malignancies 33-35. The downregulation of the ALKBH5 remarkably decreases the mRNA levels in the cytoplasm, which indicated that ALKBH5 primarily affects mRNA export and RNA metabolism 36.

m^6^A RNA binding proteins (RBPs) called m^6^A “readers” participate in the specific recognition of m^6^A‑modified targeted RNA, thereby triggering the downstream biological events 37, 38. YT521-B homology (YTH) domain family proteins (YTHDFs) and YTH domain-containing proteins (YTHDCs) belong to these proteins 39. By interacting with the initiation factors, YTH domain family proteins 1 (YTHDF1) modulates the translation initiation machinery to augment the translation efficiency of target RNAs 6. YTH domain family proteins 2 (YTHDF2) can increase the degradation of many target mRNA 2. YTHDF2 mediates the degradation of target RNA by recruiting the carbon catabolite repression 4 (CCR4)-negative on TATA-less (NOT) complex and interacting with the SH domain of CNOT1 via its N-terminal region 40. YTH domain family proteins 3 (YTHDF3) interacts with YTHDF1 to accelerate the translation of methylated mRNAs and also contributes to mRNA decay induced by YTHDF2. This indicates a complex cooperative mechanism between YTHDF proteins 41. Other reader proteins that can identify the m^6^A motif include the heterogeneous nuclear ribonucleoproteins (hnRNP) family (hnRNPA2B1, hnRNPC, and hnRNPG) 42, 43 and insulin-like growth factor 2 mRNA-binding proteins (IGF2BPs, including IGF2BP1/2/3) 44, 45 (Figure 2).

### 1.2 Characteristics, regulatory mechanisms, and biological functions of autophagy

**Figure 3:**
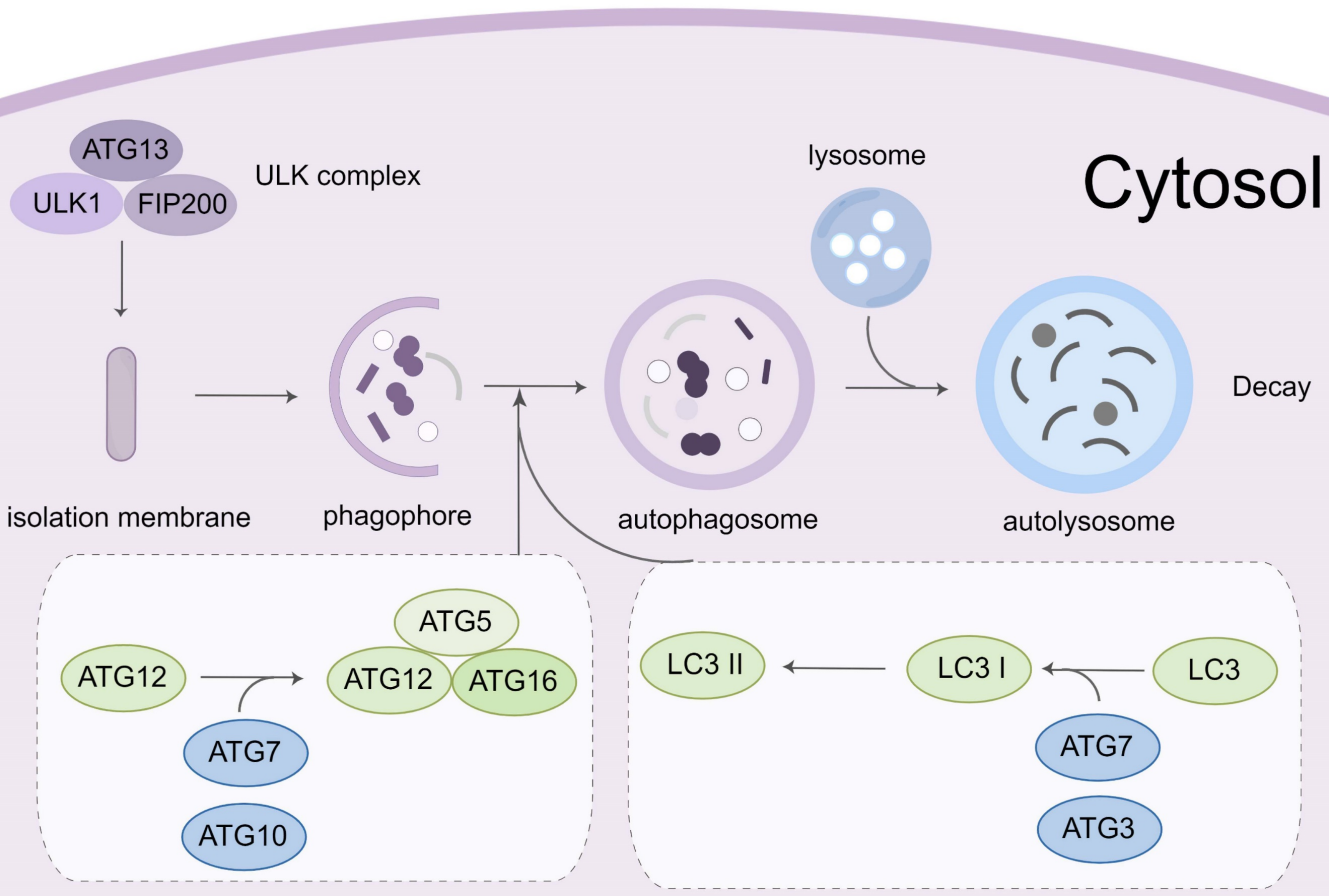
** Molecular mechanisms of autophagy process.** Autophagy initiation begins with the activation of the ULK1 complex under the control of AMPK and mTORC1. Induction of the ULK1 complex transfers the PI3K class III complex from the cytoplasm to the pre-autophagosomal structure, thereby promoting phagophore formation. The phagophore continues to expand, close, and form autophagosomes through the action of two ubiquitin-like conjugation systems: (1) the ATG12 system and (2) the LC3 system. Subsequently, the outer membrane of the autophagosome fuses with the lysosomal membrane to form autolysosomes where the cargo degradation occurs. UVRAG, RAB7A, and LAMP2 mediate autophagosome maturation and fusion with lysosomes. AMPK, 5' adenosine monophosphate-activated protein kinase; ULK1, Unc-51 Like Autophagy Activating Kinase 1; FIP200: Family interacting protein 200Kd; mTOR, mammalian target of rapamycin; PI3K, phosphoinositide 3-kinase; ATG, Autophagy related gene; LC3, Microtubule-associated protein 1A/1B-light chain 3; UVRAG, UV radiation resistance-associated gene protein; RAB7A, Ras-related protein Rab-7a; LAMP2, lysosomal-associated membrane protein 2.

Autophagy is a complicated process that requires the encapsulation of cytoplasmic components in the double-membrane vesicles and their transportation to lysosomes for destruction 46. This process begins with the Unc-51-like kinase 1 (ULK1)-autophagy related gene (ATG)13-family interacting protein 200kD (FIP200) kinase complex (namely ULK complex) 47. When the complex is exposed to environmental stress or physical and chemical damage, the ULK complex is activated cooperatively by AMP-activated protein kinase (AMPK) and ULK1. The vacuolar protein sorting (VPS)34, VPS15, ATG14, and beclin-1 proteins from the phosphatidylinositol 3-kinase (PI3K) complex, which can phosphorylate and activate the ULK complex. The formation of phagophores is mediated by the activation of the PI3K complex 48, 49. The extension of the phagophore depends on two ubiquitin-like conjugation mechanisms. The ATG12-ATG5-ATG16 complex is initially produced by the functions of ATG7 and ATG10 50. Simultaneously, microtubule-associated protein light chain 3 (LC3) is cleaved to generate soluble LC3-I by ATG4. It can conjugate to the head group of the membrane lipid phosphatidylethanolamine (PE), which is mediated by the ATG7, ATG3, and ATG12-ATG5-ATG16 complex. Autophagosomes develop and transfer to lysosomes during this time. Through the synaptosomal-associated protein 29 (SNAP29) and lysosomal vesicle-associated membrane protein (VAMP) 8, the autophagosome merges with the lysosome to form an autolysosome. Many proteins and signaling pathways are involved in this process, such as ATG12, ATG5, ULK119^51^, PI3K-AKT-mTORC1 signal pathway 52, and the AMPK pathway (Figure 3).

## 2. Role of m^6^A methylation in the regulation of autophagy

### 2.1 Regulatory effect of m^6^A methyltransferases on autophagy

#### 2.1.1 METTL3

**Figure 4:**
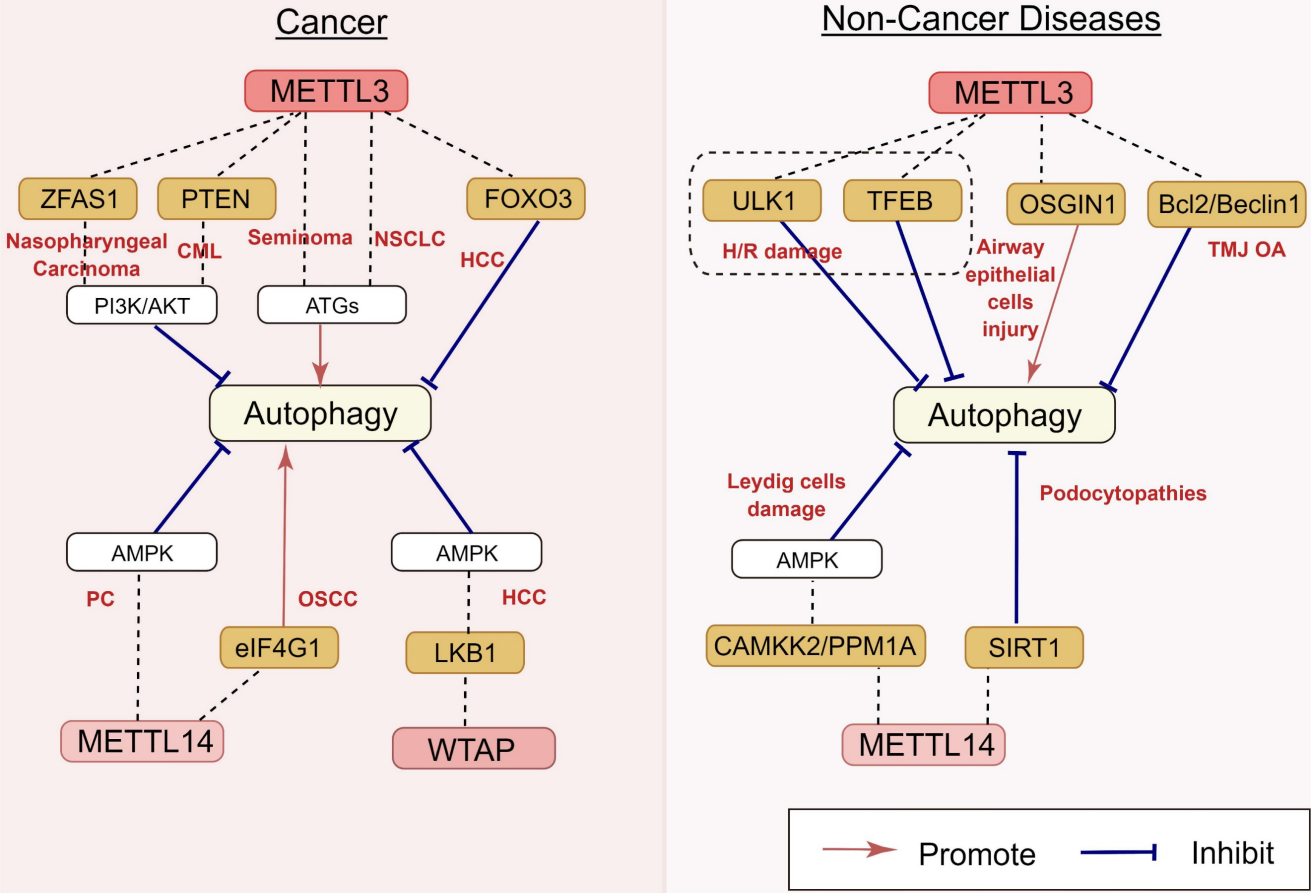
** Regulatory effects of m^6^A methyltransferases on autophagy in human diseases.** m^6^A methyltransferases play an important role in nasopharyngeal carcinoma, CML, seminoma, NSCLC, HCC, PC, OSCC, H/R damage, airway epithelial cells injury, TMJ OA, Leydig cells damage, and podocytopathies, by targeting ZFAS1, PTEN, FOXO3, eIF4G1, LKB1, ULK1, TFEB, OSGIN1, Bcl2/Beclin1, CAMKK2, PPM1A, and SIRT1. The underlying mechanisms involve the activation of the PI3K/AKT pathway, AMPK and ATGs' activation. CML: chronic myeloid leukemia; NSCLC: non-small cell lung cancer; HCC: hepatocellular carcinoma; PC: pancreatic carcinoma; OSCC: oral squamous cell carcinoma; H/R: hypoxia/reoxygenation; TMJ OA: temporomandibular joint osteoarthritis.

As the key m^6^A methyltransferase, the expression of METTL3 is closely associated with the occurrence and development of many cancers by targeting autophagy (Figure 4). In hepatocellular carcinoma (HCC) cells, the depletion of METTL3 can promote autophagy by inducing autophagy-related gene transcription and leading to forkhead box protein O3 (FOXO3) degradation via a YTHDF1-dependent pathway 53. Similarly, in seminoma, the overexpression of METTL3 resulted in the upregulation of the ATG5 gene with the increase of ATG5 m^6^A level in TCam-2 cells, thus promoting autophagy 54. Guo et al. 55 reported that METTL3 induces autophagy in non-small cell lung cancer (NSCLC) by upregulating the expression of LC3B, ATG5, and ATG7.

The correlation between m^6^A epigenetic regulation and autophagy is also found in hematological tumors. A recent study showed that lncRNA-00470 decreases the phosphatase and tensin homolog (PTEN) stability by triggering METTL3-mediated m^6^A modification, thus inhibiting cell autophagy while promoting chemoresistance in chronic myeloid leukemia (CML) 56. Furthermore, METTL3 also contributes to the regulation of autophagy in benign disorders. In the mouse model with temporomandibular joint osteoarthritis (TMJ OA), METTL3 inhibited the apoptosis and autophagy of chondrocytes induced by tumor necrosis factor-α (TNF-α) stimulation in vitro 57. Chen et al. 58 reported that METTL3 overexpression can decrease the RNA stability and expression of ATG7 via m^6^A modification, impairing autophagy in osteoarthritis (OA) fibroblast-like synoviocytes (FLSs). A previous study showed that the upregulation of METTL3 induced by lipotoxicity promotes rubicon expression in an m^6^A-dependent manner, inhibiting autophagy and further suppressing the clearance of lipid droplets (LDs) via lysosomes in nonalcoholic fatty liver disease (NAFLD) 59. However, the regulatory role of METTL3 in autophagy is still controversial. Yuan et al. reported that particulate matter 2.5 (PM_2.5_)-induced METTL3 upregulation can maintain oxidative the stability of stress induced growth inhibitor 1 (OSGIN1) mRNA by mediating m^6^A modification, thereby activating autophagy in air pollution-induced human airway epithelial cell injury 60 (Figure 4). These disparate conclusions in the field suggest that METTL3 can play more complicated roles in autophagy regulation, which will be an interesting area for research in the future.

#### 2.1.2 METTL14

METTL14 is another key component of the methyltransferase complex. It is involved in the regulation of autophagy (Figure 4). METTL14 is upregulated in pancreatic cancer, and the downregulation of METTL14 sensitizes pancreatic cancer cells to cisplatin by activating autophagy 61. The overexpression of METTL14 promoted autophagy by decreasing eukaryotic translation initiation factor 1 (eIF4G1) mRNA expression, thereby inhibiting the migration, invasion, and proliferation of oral squamous cell carcinoma (OSCC) cells 62. Lu et al. reported that podocyte injury upregulated the expression of METTL14, resulting in the degradation of silencing information regulator 2 related enzymes 1 (SIRT1) mRNA via m^6^A modification. The downregulation of METTL14 can increase SIRT1 mRNA levels by inhibiting m^6^A modification of SIRT1 mRNA, resulting in autophagy activation in podocytes and consequently alleviating proteinuria and delaying the progression of podocytopathies 63.

#### 2.1.3 WTAP

WTAP is also involved in autophagy regulation (Figure 4). In HCC, WTAP expression is upregulated. WTAP can increase the m^6^A modification of liver kinase B1 (LKB1) mRNA, decrease the stability of LKB1 transcripts, and downregulate its expression. As an upstream target of AMPK, the decrease of LKB1 can inhibit the phosphorylation of AMPK and damage the autophagy of liver cancer to a certain extent 64. m^6^A methyltransferases for autophagy regulation are presented in Table 1.

### 2.2 Regulatory effect of m^6^A demethylases on autophagy

#### 2.2.1 FTO

**Figure 5:**
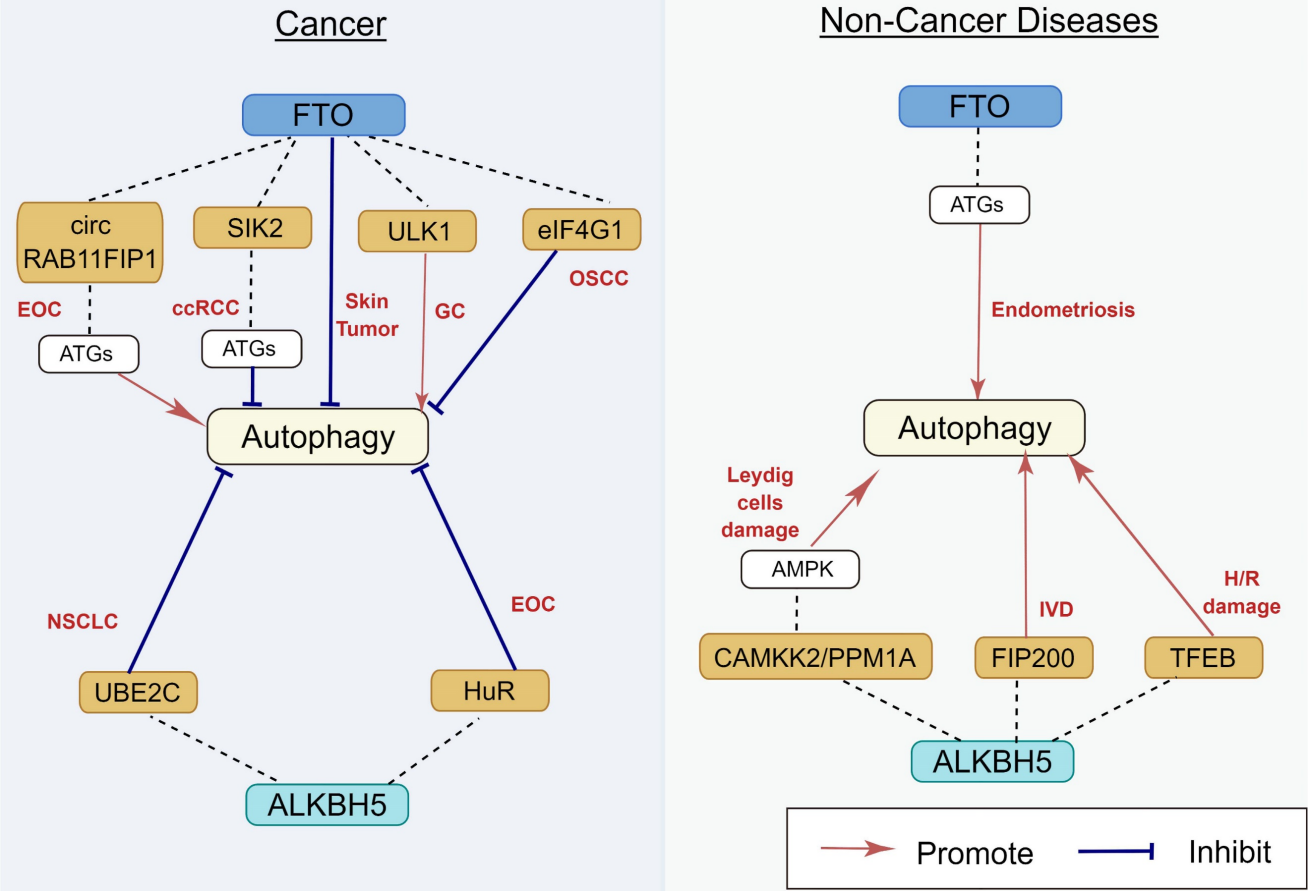
** Regulatory effect of m^6^A demethylases on autophagy in human diseases.** m^6^A demethylases play an important role in EOC, ccRCC, skin tumor, GC, OSCC, NSCLC, endometriosis, TMJ OA, Leydig cells damage, IVD, and H/R damage, by targeting circ RAB11FIP1, SIK2, eIF4G1, ULK1, UBE2C, HuR, TFEB, FIP200, CAMKK2, and PPM1A. The underlying mechanisms involve the activation of AMPK and ATGs. EOC: epithelial ovarian cancer; ccRCC: clear cell renal cell carcinoma; GC: gastric carcinoma; OSCC: oral squamous cell carcinoma; NSCLC: non-small cell lung cancer; IVD: intervertebral disc degeneration; H/R: hypoxia/reoxygenation.

FTO, the first reported m^6^A demethylase, is found to be frequently dysregulated in its expression and functions in many human diseases 65 (Figure 5). The relationship between FTO and autophagy was gradually studied over the last few years. Jin et al. 66 demonstrated that FTO can specifically upregulate the ULK1 protein level via m^6^A mediated demethylation, thereby promoting the activation of autophagy. Similarly, Zhang et al. also reported that FTO promotes cisplatin resistance by facilitating autophagy by targeting ULK1 via an m^6^A‐dependent manner in gastric cancer cells 67. Moreover, the downregulation of FTO suppressed the expression of ATG5 and ATG7, inhibiting autophagosome formation, thereby restaining autophagy and adipogenesis 68. In human endometriosis, FTO overexpression can promote autophagy by m^6^A modification of ATG5, which could inhibit glycolysis, proliferation, and metastasis of endometriotic stromal cells (EESCs) 69. However, previous studies have also shown that FTO can inhibit autophagy. In clear cell renal cell carcinoma (ccRCC), downregulation of FTO increases autophagic flux by targeting ATG5 and ATG7, which also impairs ccRCC growth and metastasis in vitro and vivo 70. In oral squamous cell carcinoma, after FTO knockdown, YTHDF2 binds with eIF4G1 transcripts containing m^6^A, resulting in mRNA degradation and downregulating the expression of eIF4G1 protein, thereby activating autophagy and suppressing tumor growth 71. Low-level arsenic exposure can stabilize FTO by inhibiting autophagy, whereas the increase in FTO can in turn inhibit autophagy, thus forming a positive feedback loop to maintain FTO accumulation and promote arsenic tumorigenicity 72. This discrepancy can be explained by evidence indicating that autophagy is a highly regulated and complicated event, and the status of autophagy regulated by m^6^A modification depends on the different cell types and/or on the stage of disease progression. Therefore, the detailed regulatory mechanisms of FTO on autophagy should be further elucidated.

#### 2.2.2 ALKBH5

Previous studies have shown that ALKBH5 contributes to the pathophysiology of several human diseases by increasing or inhibiting autophagy 73 (Figure 5). Furthermore, ALKBH5 can promote autophagy in many organs and tissues. Li et al. 74 reported that bone-derived mesenchymal stem cells (BMSCs) co-cultured with nucleus pulposus cells (NPCs) can increase ALKBH5 expression in the NPCs, thereby increasing autophagy by decreasing m^6^A modification of FIP200 mRNA and upregulating FIP200 expression. In hypoxia/reoxygenation (H/R)-induced cardiomyocytes, the expression of transcription factor EB (TFEB) was downregulated because of an increase in pre-mRNA m^6^A methylation. ALKBH5 can decrease the methylation of TFEB pre-mRNA, which resulted in autophagy activation 75. In Leydig cells, human chorionic gonadotropin (HsCG) increases ALKBH5 expression by increasing ALKBH5 transcription. ALKBH5 upregulation decreases m^6^A levels, which alleviates m^6^A-mediated protein phosphatase 1A, magnesium dependent, alpha isoform (PPM1A) translation and upregulates calcium/calmodulin-dependent protein kinase kinase 2(CAMKK2) expression by attenuating m^6^A-mediated mRNA degradation, which subsequently contributes to autophagy activation 76.

Furthermore, ALKBH5 can also inhibit autophagy under certain circumstances. In NSCLC, ALKBH5 can maintain the transcripts of UBE2C (a ubiquitin-binding enzyme that can catalyze protein degradation in the 26s proteasome) by eliminating the m^6^A methylation of its pre-mRNA, thereby stabilizing UBE2C and suppressing autophagy 77. In epithelial ovarian cancer, silencing of ALKBH5 promotes autophagy and inhibits the proliferation and invasion of SKOV3 cells by activating the PI3K-AKT-mTOR signaling pathway, whereas the overexpression of ALKBH5 exerts an opposite effect 15. These discrepancies can be attributed to the cell-type-specific context-dependent role of autophagy, which requires further investigation. The m^6^A demethylases for autophagy regulation are presented in Table 2.

### 2.3 Regulatory effect of m^6^A binding proteins on autophagy

**Figure 6:**
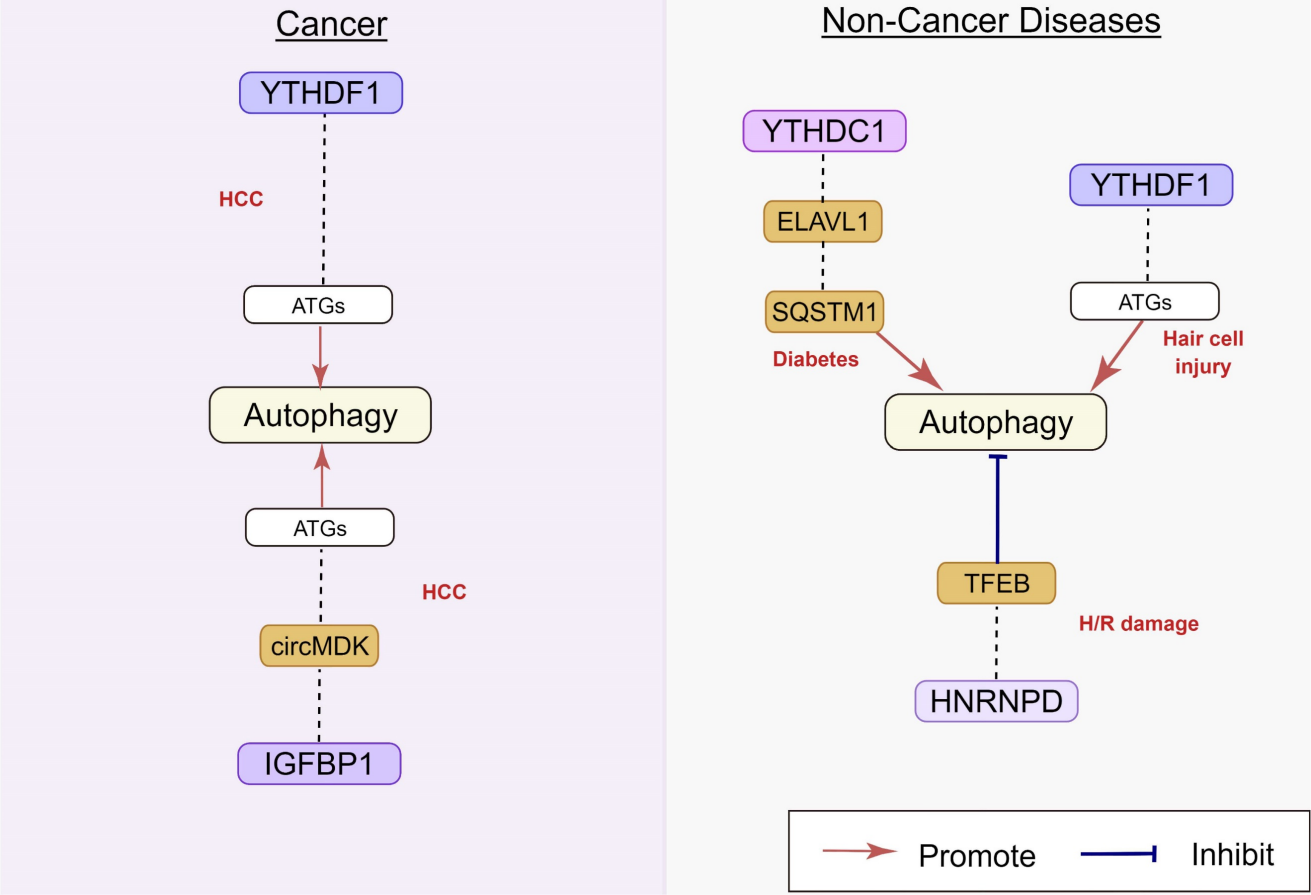
** Regulatory effect of m^6^A binding protein on autophagy in human diseases.** m^6^A binding protein play an important role in HCC, diabetes, hair cell injury, and H/R damage, by targeting circ MDK, ELAVL1, TFEB, and SQSTM1. The underlying mechanisms involve the activation of ATGs. HCC: hepatocellular carcinoma; H/R: hypoxia/reoxygenation.

m^6^A binding proteins can recognize and bind to m^6^A modification sites, controlling the modified RNA's destiny. Recently studies indicated that dysregulation of m^6^A binding proteins might lead to misinterpretation of modified target RNAs, thus affecting autophagy (Figure 6).

#### 2.3.1 YTHDF and YTHDC family

The YTHDF and YTHDC family proteins are the most important binding proteins for m^6^A modification, as they contain a YTH domain that can bind to RNA sequence motifs 78. In human hepatocellular carcinoma (HCC), hypoxia inducible factor-1α (HIF-1α) induced YTHDF1 expression was positively associated with hypoxia-induced autophagy and autophagy-related HCC progression via promoting the translation of autophagy-related genes ATG2A and ATG14 in an m^6^A-dependent manner 79. The chemotherapeutic drug cisplatin (CDP) induced YTHDF1 expression could protect sensory hair cells (HCs) against CDP-induced apoptosis by promoting the translation of autophagy-related genes ATG14, along with enhancing autophagy 80. In addition, Hao et al. recently identified the molecular mechanism of m^6^A in the regulation of starvation-induced autophagy. Their results showed that YTHDF3 promotes autophagy by recognizing the modification of m^6^A sites near the stop codon of FOXO3 mRNA. YTHDF3 also recruits eIF3a and eIF4B to facilitate FOXO3 translation, which subsequently initiates autophagy 81. Liang et al. 82 reported that the decrease in m^6^A reader YTHDC1 inhibited autophagy by accelerating sequestosome1 (SQSTM1) nuclear mRNA decay in the keratinocytes of diabetic skin, resulting in impaired migration of keratinocytes and delayed wound healing.

#### 2.3.2 IGF2BP family

Studies have shown that the lncRNA LINRIS could inhibit the degradation of the autophagy-lysosome pathway-dependent m^6^A reader IGF2BP2 by binding to the K139 ubiquitination site of IGF2BP2, maintaining MYC-mediated glycolysis and colorectal cancer cell proliferation 83. Another study showed that the lncRNA MALAT1 was a target of IGF2BP2 in NSCLC. IGF2BP2 could enhance the stabilization of MALAT1 via an m^6^A-dependent mechanism, activating its downstream target ATG12 and NSCLC proliferation 84.

#### 2.3.3 hnRNP family

hnRNPs represent a large subclass of RBPs that contribute to the multiple aspects of nucleic acid 85. A previous study has shown that hnRNPD can block METTL3-mediated autophagy by downregulating TFEB mRNA expression 75. Mechanistically, when TFEB was methylated at two m^6^A residues in the 3'-UTR by METTL3, hnRNPD could bind to TFEB pre-mRNA, decreasing TFEB mRNA levels. Li et al. found that hnRNPC was the decreased m^6^A regulator in human abdominal aortic aneurysm and a correlation analysis indicated that the level of hnRNPC was positively correlated with the infiltration degree of circulating memory T cells, macrophages, and mast cells 86.

Overall, the above findings show that diverse m^6^A binding proteins have been identified, which need to be thoroughly investigated in the future. As m^6^A binding proteins are necessary for the downstream physiological activities of m^6^A modification, the same m^6^A regulator may have opposing regulatory effects after directly binding to distinct binding proteins. To summarize, regulating the binding of m^6^A-modified RNA to "readers" might be a novel disease treatment strategy in the future. A list of m^6^A readers for autophagy regulation is provided in Table 3.

## 3. Role of autophagy in regulating m^6^A methylation

An m^6^A modification enzyme affects the level of autophagy, however, the relationship between m^6^A methylation and autophagy is not limited to this phenomenon. In parallel, autophagy activation or impairment also affects the m^6^A modification enzyme and participates in disease development. In melanoma cells 87, metabolic stress conditions, such as a model starvation medium (Hank's Balanced Salt Solution, HBSS), increased FTO mRNA and protein levels, accompanied by a decreased m^6^A level. As stress conditions can trigger autophagy, the authors reasoned that the autophagy pathway mediated the FTO increase by HBSS. Interestingly, they found that the knockdown of the autophagy key genes ATG5 or ATG7 significantly reduced HBSS-induced FTO, suggesting that autophagy-induced melanoma tumorigenesis, which was promoted by the increased FTO. Furthermore, Cui et al. 72 found that low-level arsenic could stabilize FTO by inhibiting the p62-mediated selective autophagic degradation of FTO. Their data showed that the deletion of the critical autophagy related genes ATG5 or ATG7 increased FTO stability, indicated by the increased half-life. Furthermore, increased FTO level blocked autophagy, resulting in a positive feedback loop to maintain FTO accumulation.

In summary, these findings highlight complex interactions between autophagy and m^6^A regulators. However, limited evidence is available on the role of autophagy in regulating m^6^A RNA modification. Therefore, further research is required to explore whether and how m^6^A regulators are tightly regulated by autophagy pathways in different pathological conditions, which will provide a broader perspective for treating diseases.

## 4. Potential diagnostic value of m^6^A modification in regulating autophagy

m^6^A regulators are commonly upregulated or downregulated in numerous human diseases, contributing to disease progression. Correlations between m^6^A regulators and clinical parameters have been confirmed in previous studies 88, 89. Accumulating evidence has shown that targeting m^6^A regulators and their regulatory proteins are emerging as a novel diagnostic approach.

Previous studies indicated that YTH domain-containing proteins act as potential diagnostic biomarkers in patients with cancer 90-93. Chen et al. 94 examined differentially expressed m^6^A and autophagy genes in esophageal squamous cell carcinoma (ESCC). They developed and validated a predictive model based on six characteristic autophagy genes for predicting the survival of patients with ESCC. Circulating tumor cells (CTCs) derived from tumors can truly reflect the status and progression of a tumor. A recent study showed that mass spectrometry could be used to monitor m^6^A levels in CTCs. Huang et al. demonstrated that compared with whole blood samples, m^6^A levels were significantly increased in CTCs of patients with lung cancer. This study showed that CTCs could be used as an early non-invasive diagnostic indicator of cancer 95. Another study showed the abnormal expression of m^6^A regulators in endometriosis and indicated that hnRNPA2B1 and hnRNPC might be correlated with immune response, serving as useful diagnostic biomarkers for endometriosis 96. Collectively, these findings suggest that m^6^A regulators may have the potential to be a specific and sensitive biomarker of human disease diagnosis.

## 5. Potential therapeutic value of m^6^A modification in regulating autophagy

### 5.1 Role of autophagy regulation by m^6^A in disease progression

m^6^A methylation regulates autophagy by affecting the translation or stability of several autophagy-related genes. However, the role of autophagy in the occurrence and development of human diseases cannot be generalized.

**Figure 7:**
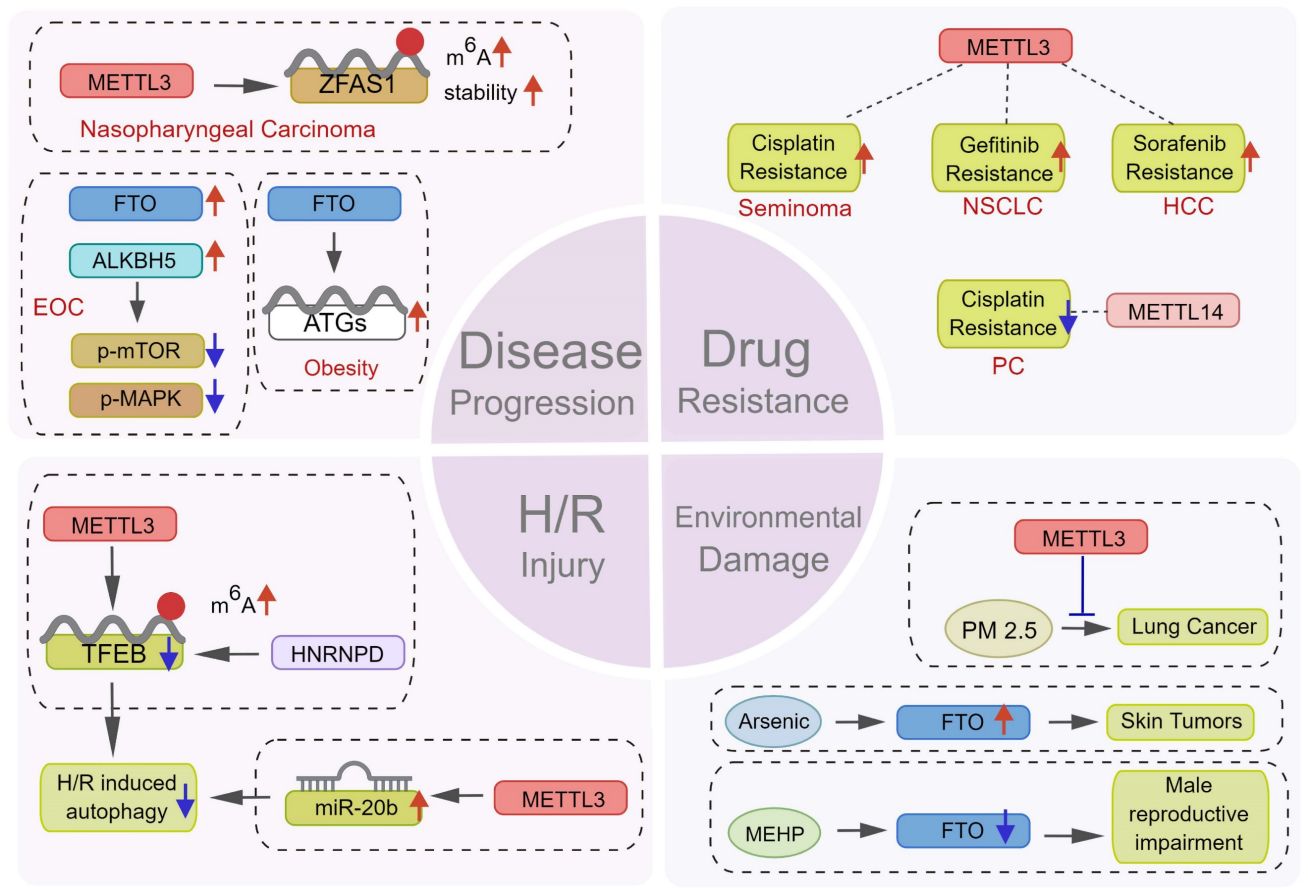
** Potential therapeutic value of m^6^A modification in regulating autophagy.** m^6^A modification plays a key role in many aspects of disease treatment. m^6^Aregulators can become a potential target for therapy by affecting (1) the progression of tumor and non-tumor diseases, (2) the sensitivity of tumor cells to various chemotherapeutic drugs, (3) the response of cells to H/R damage, and (4) the damage of environmental pollutants.

As an adaptive mechanism under stress, autophagy protects cells against pressures including hypoxia and chemotherapy, thus promoting disease progression. Peng et al. 97 found that METTL3 could increase the m^6^A level and stability of the lncRNA ZFAS1, subsequently activating autophagy via the PI3K/AKT pathway, thus promoting the proliferation and metastasis of nasopharyngeal carcinoma cells. In epithelial ovarian cancer, CircRNA-AB11FIP1 can increase demethylase FTO level and activate autophagy by altering the m^6^A level of ATG7 to promote the malignant behavior of ovarian cancer cells 98. Wang et al. 68 reported that FTO promoted autophagy and adipogenesis by directly targeting Atg5 and Atg7 transcripts and mediating their expression in an m^6^A-YTHDF2-dependent manner, thus facilitating the development of therapeutic strategies for the prevention and treatment of obesity. In contrast, as a type of programmed cell death, the abnormal activation of autophagy also results in cell death in certain circumstances 15. Recently, a study reported that downregulating ALKBH5 could increase autophagy and attenuate the proliferation and invasion potentiality of ovarian cancer cells *in vitro* and *in vivo*, whereas the ectopic expression of ALKBH5 could reverse this effect 15 (Figure 7). These findings suggest that targeting m^6^A to induce or inhibit autophagy might be a promising novel therapeutic strategy against several diseases. However, given the contradictory role of autophagy regulation by m^6^A in tumor development, the underlying mechanisms need further exploration.

### 5.2 Role of autophagy regulation by m^6^A in tumor chemotherapy resistance

Chemotherapy and targeted drug therapy are important means of tumor therapy. Accumulating evidence indicates that tumor cells can acquire resistance to death via diverse biological mechanisms, leading to anticancer drug resistance of tumors 99, 100. Recently, multiple mechanisms by which tumor cells become resistant to anticancer drugs have been discovered, among which epigenetic change 87, 101-107-mediated autophagy 108-110 also plays a critical role.

Lin et al. 53 discovered that METTL3-dependent sorafenib resistance exists in HCC and was mediated by promoting autophagy. The combination of autophagy inhibitors and sorafenib or treating HCC cells under hypoxia obtained showed a significant sensitivity to sorafenib, providing another novel strategy for treating drug-resistant liver cancer. In seminoma 54, METTL3 could increase autophagy by modulating ATG5, thus promoting cisplatin resistance. Therefore, METTL3 was a potential therapeutic target to reverse cisplatin resistance in seminoma. β-elements could reverse gefitinib resistance in NSCLC cells by inhibiting METTL3-mediated autophagy. Mechanistically, METTL3 can regulate autophagy by targeting ATG5, ATG7, LC3B, and SQSTM1 55. However, autophagy is not always conducive to chemotherapy resistance. It could also enhance sensitivity to chemotherapy. Kong et al. 61 found that the artificial regulation of METTL14 expression may significantly damage the proliferation of pancreatic cancer cells in the presence of cisplatin. METTL14 downregulation could result in autophagy activation via the mTOR signaling pathway, thus effectively improving cisplatin sensitivity in pancreatic cancer cells (Figure 7).

In summary, m^6^A modification regulates chemotherapy resistance by affecting various factors in different tumors, with autophagy as a downstream event. As drug resistance in cancers is becoming more common, targeted m^6^A modification provides a more accurate and individualized treatment option. However, the mechanism of m^6^A modification to regulate autophagy in tumor chemotherapy resistance is complex. This may be attributed to the heterogeneity of m^6^A modification enzymes in different tumors, and the “double-sword” effect of autophagy on drug resistance regulation. Thus, the mechanism underlying m^6^A modification in chemotherapy resistance needs further elucidation.

### 5.3 Role of autophagy regulation by m^6^A in hypoxia-reperfusion injury

Ischemia and reperfusion (I/R) injury often occurs during and after surgery, which may cause serious, even life-threatening, organ damage 111. Previous studies have shown a significant difference between myocardial m^6^A levels in the ischemic myocardium and non-ischemic areas 112. Autophagy is also involved in the occurrence and development of ischemic heart disease 113. Song H et al. 75 found increased m^6^A modification in cardiomyocytes treated with H/R and I/R. METTL3 methylates the m^6^A residue of TFEB in the 3'-UTR, thus promoting the binding of the RBP hnRNPD to TFEB pre-mRNA and reducing TFEB mRNA levels. With TFEB as a medium, METTL3 reduced autophagy and damaged cardiomyocytes. METTL3 knockout can effectively improve the activity of cardiomyocytes treated with H/R. Similarly, under H/R conditions, propofol post-treatment prevented autophagy and cell death induced by H/R via the METTL3/miR-20b/ULK1 signaling pathway 114 (Figure 7). Overall, these studies showed that m^6^A regulators participating in autophagy in ischemic diseases may act as potential therapeutic targets which need further investigation.

### 5.4 m^6^A methylation mediated autophagy confers environmental damage

Nowadays, potential environmental pathogenic factors gradually enter people's line of sight. Environmental pollutants including dust, heavy metals, and plastic derivatives can damage human systems including respiration, digestion, and reproduction 115, 116. Many studies show that autophagy regulated by m^6^A also plays a potential protective role in the above-mentioned processes.

As a widely used plasticizer, the toxicity of di-(2-ethylhexyl) phthalate (DEHP) has been reported 117, 118. Zhao et al. 119 found that mono-(2-ethylhexyl) phthalate, the main metabolite of DEHP in the body, promoted m^6^A modification by decreasing FTO levels, leading to Leydig cell damage. Long-term low-level arsenic exposure could enhance FTO stability and decrease m^6^A levels by impairing selective autophagy, thus inducing the malignant transformation and tumorigenesis of keratinocytes 72. In addition, studies showed that the air pollutant PM_2.5_ could activate autophagy and promote the damage of the respiratory epithelium and the development of lung cancer 60, 120. METTL3 knockout could attenuate PM_2.5_-induced apoptosis and autophagy and protect the respiratory system (Figure 7). Thus, m^6^A methylation can regulate autophagy or protect the human body from pathogenic environmental factors considering the above-mentioned results. However, the underlying mechanisms should be further studied in depth.

## 6. Conclusions and future directions

m^6^A modification, the most prevalent post-transcriptional epigenetic mechanism, is widely distributed in eukaryote RNAs. Many studies have indicated that alterations in m^6^A modification and autophagy affect the development and progression of various human diseases, and they have important implications in the diagnosis and treatment of several human diseases. This review focuses on mutual interactions between m^6^A regulators and autophagy and the combined effect of various m^6^A regulators and autophagy on different human diseases. m^6^A regulators could modulate autophagy processing, whereas autophagy activation or inhibition could degrade m^6^A regulator to change m^6^A levels. This evidence enhances the understanding of the pathogenesis of different types of human diseases. However, whether and how m^6^A regulators and m^6^A precisely modulate autophagy remain controversial. For instance, METTL14 inhibits autophagy in testicular tissues and pancreatic cancer, whereas METTL3 promotes autophagy in the heart, liver, and endothelial cells. Similarly, METTL3 suppresses autophagy in the lungs of patients with NSCLC. The distinction between different autophagy-mediating enzymes is more evident for m^6^A demethylase. FTO can induce autophagy in various tumor tissues and cell lines, including gastric cancer, ovarian cancer, and renal cell carcinoma. Two main reasons that probably contribute to this discrepancy are as follows. Firstly, m^6^A levels, which play diverse roles in various physiological statuses, differ in different cells and/or disease progression stages 37, 121. Second, the function of autophagy is context-dependent and highly affected by the disease status and exposure to external stimuli 122, 123. Hence, more research is warranted to investigate the functions and molecular regulatory mechanisms of the association between m^6^A regulators and autophagy.

The critical role of m^6^A regulators in disease initiation and progression provides new possibilities for the early diagnosis and treatment of several human diseases. m^6^A regulators may serve as a potential non-invasive diagnostic biomarker and be used for the diagnosis and prognosis of human diseases. Altered total m^6^A levels and abnormal m^6^A regulator expression in different human diseases have been recently reported. However, their specific roles in disease diagnosis need further exploration. Besides, antibody-based m^6^A detection technology has poor specificity 124. Although various antibody-free methods have been developed to detect m^6^A sites, these methods have certain limitations such as low reproducibility, thus requiring further improvement 125-127. Moreover, m^6^A regulators whether dysregulation regulators can be considered a potential biomarker to detect early-stage diseases, and whether the total m^6^A level can be a credible predictive biomarker for monitoring treatment efficacy have not been clarified 128-130. Therefore, further research is required before applying m^6^A regulators to disease diagnosis.

Accumulating evidence indicates m^6^A modification as a new therapeutic target for disease treatment. Therefore, developing potent and specific small molecule inhibitors/activators for m^6^A regulatory proteins is crucial. In 2012, Chen et al. 131 discovered rhein, the first identified FTO inhibitor, which could alter the m^6^A levels of mRNAs inside cells, acting as a competitive inhibitor of FTO. However, rhein also biochemically inhibited demethylase ALKBH2 activity, suggesting that rhein is not an FTO-specific inhibitor. The ethyl ester form of meclofenamic acid (MA) was identified as a selective chemical inhibitor of FTO that increases m^6^A levels in the mRNA of HeLa cells in 2015^132^. Further studies showed that treating glioblastoma stem cells (GSCs) with the MA attenuated GSC-triggered tumorigenesis in GSC-engrafted mice 133. Moreover, another study indicated that R-2-hydroxyglutarate produced at high levels by mutant isocitrate dehydrogenase 1/2 enzymes exhibited anticancer effects by suppressing FTO activity, thereby increasing m^6^A levels in acute myeloid leukemia (AML) cells 134. Recently, Yankova et al. 135 reported that STM2457, a specific METTL3 inhibitor, could be used as a promising therapeutic drug for AML, which is expected to enter clinical trials as the first epigenetic inhibitor drug. However, whether m^6^A inhibitors or activators cause unpredictable side effects is unclear. Therefore, large-scale, multicenter, and collaborative clinical trials will help better elucidate the role of m^6^A modification in autophagy and the potential molecular mechanism of disease development, thus providing novel biomarkers and therapeutic targets for human diseases.

Taken together, with the development of Bio-Technology, the detailed regulatory mechanisms underlying the interaction networks between m^6^A modification and autophagy will be more extensively investigated. And more diagnostic and therapeutic targets of m^6^A regulators that contribute to human disease progression will be further explored.

## Figures and Tables

**Table 1:** Regulatory effect of m^6^A methyltransferases and their molecular mechanisms

m^6^A component	Related gene	Diseases	m^6^A level	Association between m^6^A and autophagy*	Autophagy regulated mechanism	References
METTL3	FOXO3	Hepatocellular carcinoma	Decrease	Negative	FOXO3	53
METTL3	Bcl2	Temporomandibular joint osteoarthritis	Decrease	Negative	Bcl2	57
METTL3	miR-20b	Hypoxia/reoxygenation-treated endothelial cell damage	-	Negative	ULK1	114
METTL3	TFEB	Hypoxia/reoxygenation-treated cardiomyocyte damage	Increase	Negative	AMPK pathway	75
METTL3	PTEN	Chronic myelocytic leukaemia	Increase	Negative	PI3K/AKT pathway	56
METTL3	LC3B, ATG5, ATG7	Non-small-cell lung cancer	Increase	Positive	LC3B, ATG5, ATG7	55
METTL3	ATG5	Seminoma	Increase	Positive	ATG5	54
METTL3	OSGIN1	Pollution-induced airway epithelial cell injury	Increase	Positive	OSGIN1	60
METTL3	ZFAS1	Nasopharyngeal carcinoma	Increase	Positive	PI3K/AKT pathway	97
METTL14	SIRT1	Podocytopathies	Increase	Negative	SIRT1	63
METTL14	AMPK1/2, ERK12	Pancreatic cancer	Increase	Negative	mTOR pathway	61
METTL14	CAMKK2, PPM1A	Testosterone synthesis disorder	Decrease	Negative	AMPK pathway	76
METTL14	eIF4G1	Oral squamous cell carcinoma	Increase	Positive	-	62
WTAP	LKB1	Hepatocellular carcinoma	Increase	Negative	AMPK pathway	64

*Negative indicates that the increasing m^6^A level of RNA can inhibit autophagy, while positive indicates that it can promote autophagy.

**Table 2:** Regulatory effect of m^6^A demethylases and molecular mechanisms

m^6^A component	Related gene	Diseases	m^6^A level	Association between m^6^A and autophagy*	Autophagy regulated mechanism	References
FTO	ULK1	-	-	Positive	ULK1	66
FTO	SIK2	Clear cell renal cell carcinoma	Decrease	Positive	ATG5, ATG7	70
FTO	ATG5, ATG7	Squamous cell carcinoma	Decrease	Positive	m TOR/TFEB/AMPK	72
FTO	eIF4G1	Oral squamous cell carcinoma	-	Positive	eIF4G1	71
FTO	ATG5, ATG7	Obesity	Decrease	Negative	ATG5, ATG7	68
FTO	ULK1	Gastric cancer	Decrease	Negative	ULK1	67
FTO	ATG5, ATG7	Epithelial ovarian cancer	Decrease	Negative	ATG5, ATG7	98
FTO	ATG5	Endometriosis	Increase	Negative	ATG5	69
ALKBH5	CAMKK2, PPM1A	Testosterone synthesis disorder	Increase	Negative	AMPK pathway	76
ALKBH5	FIP200	Intervertebral disc degeneration	-	Negative	ULK1 complex	74
ALKBH5	TFEB	Hypoxia/reoxygenation-treated cardiomyocytes	Increase	Negative	AMPK pathway	75
ALKBH5	UBE2C	Non-small cell lung cancer	Decrease	Positive	LC3B, ATG5, ATG7	77
ALKBH5	HuR, Beclin1&Bcl-2	Epithelial ovarian cancer	Decrease	Positive	EGFR/PIK3C/AKT/mTOR pathway	15

*Negative indicates that the increasing m^6^A level of RNA can inhibit autophagy, while positive indicates that it can promote autophagy.

**Table 3:** Regulatory effect of m^6^A binding proteins and molecular mechanisms

m^6^A component	Related gene	Diseases	m^6^A binding protein level	Association between m^6^A binding protein and autophagy*	Autophagy regulated mechanism	References
YTHDF1	ATG2A, ATG14	Hepatocellular carcinoma	Increase	Positive	ATG2A, ATG14	79
YTHDF1	ATG14	Hair cell injury	Decrease	Positive	ATG14	80
YTHDF3	FOXO3	-	-	Positive	ATGs	81
YTHDC1	SQSTM1	Diabetic skin injury	Decrease	Positive	SQSTM1	82
IGF2BP2	MALAT1	Non-small cell lung cancer	Increase	Positive	ATG12	84
hnRNPD	TFEB	Hypoxia/reoxygenation-treated cardiomyocytes	Increase	Negative	AMPK pathway	75

*Negative indicates that the increasing m^6^A level of RNA can inhibit autophagy, while positive indicates that it can promote autophagy.

## Abbreviations

m6A: N^6^-methyladenosine
UV: ultraviolet
METTL3: methyltransferase-like 3
METTL14: methyltransferase-like 14
WTAP: wilms tumor 1-associated protein
ALKBH5: human AlkB Homolog H5
FTO: fat mass and obesity-associated protein
RBP: RNA binding proteins
YTH: YT521-B homology
YTHDF: YTH domain family proteins
YTHDC: YTH domain-containing protein
CCR4: carbon catabolite repression 4
NOT: negative on TATA-less
HNRNP: heterogeneous nuclear ribonucleoproteins
IGF2BP: insulin-like growth factor 2 mRNA-binding proteins
ULK1: unc-51-like kinase 1
ATG: autophagy related gene
FIP200: Family interacting protein 200kD
AMPK: AMP-activated protein kinase
VPS34/15: vacuolar protein sorting 34/15
PI3K: phosphatidylinositol 3-kinase
LC3: microtubule-associated protein light chain 3
PE: phosphatidylethanolamine
SNAP29: synaptosomal-associated protein 29
VAMP: vesicle-associated membrane protein
HCC: hepatocellular carcinoma
FOXO3: forkhead box protein O3
PTEN: phosphatase and tensin homolog
CML: chronic myeloid leukemia
NSCLC: non-small cell lung cancer
TNF-α: tumor necrosis factor-α
TMJ OA: temporomandibular joint osteoarthritis
FLS: fibroblast-like synoviocytes
NAFLD: nonalcoholic fatty liver disease
LDs: lipid droplets
PM2.5: particulate matter 2.5
OSGIN1: oxidative stress induced growth inhibitor 1
eIF4G1: eukaryotic translation initiation factor 1
OSCC: oral squamous cell carcinoma
SIRT1: silencing information regulator 2 related enzyme 1silencing information regulator 2 related enzyme 1
LKB1: liver kinase B1
EESC: endometriotic stromal cells
ccRCC: clear cell renal cell carcinoma
BMSC: bone-derived mesenchymal stem cells
NPC: nucleus pulposus cells
TFEB: transcription factor EB
H/R: hypoxia/reoxygenation
HsCG: human chorionic gonadotropin
PPM1A: protein phosphatase 1A
CAMKK2: calcium/calmodulin-dependent protein kinase kinase 2
UBE2C: Ubiquitin-Conjugating Enzyme E2 C
HIF-1α: hypoxia inducible factor-1α
CDP: chemotherapeutic drug cisplatin
HCs: hair cells
SQSTM1: sequestosome1
hnRNPs: heterogeneous nuclear ribonucleoproteins
AAA: abdominal aortic aneurysm
HBSS: Hank's Balanced Salt Solution
ESCC: esophageal squamous cell carcinoma
CTC: circulating tumor cells
EOC: epithelial ovarian cancer
mTOR: mechanistic target of rapamycin
I/R: Ischemia and reperfusion
DEHP: Di-(2-ethylhexyl) phthalate
MEHP: mono-(2-ethylhexyl) phthalate
MA: meclofenamic acid
GSC: glioblastoma stem cell
R-2HG: R-2-hydroxyglutarate
IDH1/2: isocitrate dehydrogenase 1/2
AML: acute myeloid leukemia
